# H19 knockdown suppresses proliferation and induces apoptosis by regulating miR-148b/WNT/β-catenin in ox-LDL -stimulated vascular smooth muscle cells

**DOI:** 10.1186/s12929-018-0418-4

**Published:** 2018-02-07

**Authors:** Lei Zhang, Hailing Cheng, Yuxia Yue, Shuangzhan Li, Daping Zhang, Ruili He

**Affiliations:** 1 Department of Cardiology, Huaihe Hospital of Henan University, No.8, Baobei Road, Gulou District, Kaifeng, 475000 China; 2 Department of Obstetrics and Gynecology, Huaihe Hospital of Henan University, Kaifeng, 475000 China

**Keywords:** lncRNA, H19, Atherosclerosis, miR-148b, WNT1, WNT/β-catenin signaling

## Abstract

**Background:**

Long non-coding RNAs (lncRNAs) have been identified as critical regulators in the development of atherosclerosis (AS). Here, we focused on discussing roles and molecular mechanisms of lncRNA H19 in vascular smooth muscle cells (VSMCs) progression.

**Methods:**

RT-qPCR assay was used to detect the expression patterns of H19 and miR-148b in clinical samples and cells. Cell proliferative ability was evaluated by CCK-8 and colony formation assays. Cell apoptotic capacity was assessed by apoptotic cell percentage and the caspase-3 activity. Bioinformatics analysis, luciferase and RNA immunoprecipitation (RIP) assays were employed to demonstrate cell percentage and the relationship among H19, miR-148b and wnt family member 1 (WNT1). Western blot assay was performed to determine expressions of proliferating cell nuclear antigen (PCNA), ki-67, Bax, Bcl-2, WNT1, β-catenin, C-myc and E-cadherin.

**Results:**

The level of H19 was increased and miR-148b expression was decreased in human AS patient serums and oxidized low-density lipoprotein (ox-LDL)-stimulated human aorta vascular smooth muscle cells (HA-VSMCs). H19 knockdown suppressed proliferation and promoted apoptosis in HA-VSMCs following the treatment of ox-LDL. H19 inhibited miR-148b expression by direct interaction. Moreover, miR-148b inhibitor could reverse the effects of H19 depletion on proliferation and apoptosis in ox-LDL-stimulated HA-VSMCs. Further mechanical explorations showed that WNT1 was a target of miR-148b and H19 acted as a competing endogenous RNA (ceRNA) of miR-148b to enhance WNT1 expression. Furthermore, miR-148 inhibitor exerted its pro-proliferation and anti-apoptosis effects through activating WNT/β-catenin signaling in ox-LDL-stimulated HA-VSMCs.

**Conclusion:**

H19 facilitated proliferation and inhibited apoptosis through modulating WNT/β-catenin signaling pathway via miR-148b in ox-LDL-stimulated HA-VSMCs, implicating the potential values of H19 in AS therapy.

## Background

Cardiovascular diseases (CVD) are primary causes of death with more than 17.3 million mortality per year worldwide [1]. Atherosclerosis (AS), a major underlying basis of heart attacks and stroke, accounts for a large proportion in cardiovascular diseases. AS is an inflammatory disease with the characteristics of lipid accumulation, fibrous cap formation and necrotic core generation [2]. Multiple cell types such as smooth muscle cells (SMCs), endothelial cells (ECs) and macrophages are implicated in atherosclerotic lesion formation [3]. Moreover, previous studies showed that abnormal proliferation, apoptosis and migration of vascular smooth muscle cells (VSMCs) was closely associated with AS progression [4, 5]. Oxidized low-density lipoprotein (ox-LDL), a critical risk factor in AS progression, was reported to be able to affect VSMCs apoptosis, proliferation and invasion [6]. Thus, in the present study, we employed ox-LDL-stimulated VSMCs as an AS cell model to investigate the regulatory mechanisms involved in AS.

Long noncoding RNAs (lncRNAs), a group of noncoding RNAs with the length longer than 200 nucleotides (nt), have been identified as vital regulators in many complex diseases, including AS [7, 8]. AS-related lncRNAs have gained wide attentions due to their regulatory effect in lipid metabolism, inflammatory response, cell proliferation, apoptosis, adhesion and migration [9]. For instance, lncRNA p21 expression was markedly decreased in atherosclerotic plaques of ApoE^−/−^ mice, and lncRNA-p21 suppressed proliferation and facilitated apoptosis in VSMCs and mouse mononuclear macrophage cells via increasing p53 activity [10]. Knockdown of lncRNA RNCR3 accelerated the development of AS via blocking proliferation and migration, and inducing apoptosis of ECs and VSMCs in vitro [11]. LncRNA H19, a noncoding RNA transcript of H19 gene, has been well identified as a vitally imprinted gene in cell growth and differentiation [8]. There have been documents demonstrating the correlation between H19 and coronary artery disease risk in a Chinese population [12, 13]. Also, previous studies showed that H19 was highly expressed in human atherosclerotic lesions and rat VSMCs after injury [14, 15]. Moreover, H19 overexpression resulted in a remarkable enhancement of proliferation and a marked inhibition of apoptosis in human umbilical vein endothelial cells (HUVECs) and VSMCs [16]. However, it is still necessary to further explore the detailed molecular mechanism of H19 in AS development.

MicroRNAs, a class of small noncoding RNA about 22 nt, are implicated in the regulation of genes at posttranscriptional levels [17]. In AS, lots of microRNAs have been confirmed as vital modulators of pathological processes, such as cholesterol and lipid biosynthesis, lipoprotein metabolism and cholesterol efflux, immune responses, and endothelial cell biology and vascular function [18]. MiR-148b, located at human chromosome 12q13.13, has been identified as a tumor suppressor in some cancers such as pancreatic cancer [19], non-small cell lung cancer [20] and hepatocellular cancer [21]. Zhang et al. also verified that miR-148b overexpression suppressed proliferation and migration of VSMCs through directly targeting HSP90 in AS [22].

In our study, we aimed to further explore the underlying roles and molecular basics of H19 and miR-148b in VSMCs progression to discover potential therapy targets of AS.

## Methods

### Clinical samples

Our study was performed with the approval of Ethical Committee in Huaihe Hospital and the informed consents from all participants (50-70 years old, 30% female). Blood samples (10 ml) from AS patient (*n* = 40) without any treatment and healthy volunteers (n = 40) were collected in centrifuge tubes without any anticoagulant in our hospital. The healthy volunteers were registered referring to the standards that none of them has suffered from AS disease, malignancies and inflammatory diseases, autoimmune diseases, or recent infection (< 1 month). After collection, blood samples were placed at room temperature for about 1 h, followed by the extraction of serums via centrifugation at the speed of 3000 rpm for 5 min. Then RNA in serums was isolated using TRIzol reagent (Invitrogen, Carlsbad, CA, USA).

### Cell culture

Human aorta vascular smooth muscle cells (HA-VSMCs) were purchased from American Type Culture Collection (ATCC, Manassas, VA, USA) and maintained in F-12 K medium (ATCC) supplemented with 10% FBS (Invitrogen), 0.05 mg/ml ascorbic acid (Sigma-Aldrich, St. Louis, MO, USA), 0.01 mg/ml insulin (Sigma-Aldrich), 0.01 mg/ml transferrin (Sigma-Aldrich), 0.03 mg/ml endothelial cell growth supplement (Cell application, Santiago, CA, USA), 10 ng/ml sodium selenite (Sigma-Aldrich), 10 mM HEPES (Sigma-Aldrich), and 10 mM TES (Sigma-Aldrich) in a humidified incubator with 5% CO2 at 37 °C.

### Cell transfection and treatment

The full length sequence of H19 was amplified by PCR and then subcloned into pcDNA3.1 vectors (Invitrogen) to generate pcDNA-H19 overexpression plasmids. Small interference RNA (siRNA) targeting H19 (si-H19#1 and si-H19#2) and its negative control (si-con), siRNA against WNT1 (si-WNT1) and its scramble control (si-con), miR-148b mim**i**c (miR-148b) and its negative control (miR-con), miR-148b inhibitor (anti-miR-148b) and its negative control (anti-miR-con) were designed and synthesized by GenePharma Co. ltd (Suzhou, China). Cell transfection was performed using lipofectamine 2000 reagent (Invitrogen) following the protocol of manufacturer. To observe the effect of ox-LDL (Biosynthesis Biotechnology Company, Beijing, China) on the expression H19 and miR-148b, HA-VSMCs were treated with different doses of ox-LDL (0, 25, 50 and 75 μg/ml) for 24 h. β-catenin inhibitor XAV939 was purchased from Sigma-Aldrich.

### RT-qPCR assay

Total RNA was extracted using TRIzol® reagent (Invitrogen) referring to the manufacturer’s instructions. Equal weight RNA (1 μg) was reversely transcribed into cDNA first strand with M-MLV reverse transcriptase (Invitrogen) and random primers (H19 and β-actin) or specific reverse transcription primers (miR-148b and U6 snRNA). Then the SYBR Green Realtime PCR Master Mix (TOYOBO, Osaka, Japan) and quantitative PCR primers were employed to detect expressions of H19, β-actin, miR-148b and U6 snRNA. MiR-148b and U6 snRNA primers (reverse transcription primers and quantitative PCR primers) were synthesized by Ribobio Co., Ltd. (Guangzhou, China). The quantitative PCR primers of H19 and β-actin were listed as follows: H19, 5’-CGCTTTTGAACCAGCAGGG-3′ (forward) and 5’-TTCCCGAGGCTTTGGTGTG-3′ (reverse); β-actin 5’-AGGGGCCGGACTCGTCATACT-3′ (forward) and 5’-GGCGGCACCACCATGTACCCT-3′ (reverse). β-actin acted as an endogenous control of H19 and U6 snRNA performed as an endogenous control of miR-148b.

### Western blot assay

Whole proteins were extracted using RIPA lysis buffer (Beyotime) and then quantified using a Pierce BCA Protein Assay Kit (Thermo Fisher Scientific, Rockford, IL, USA). Equal proteins (50 μg) were separated via SDS-PAGE gel and transferred onto PVDF membranes (Millipore, Billerica, MA, USA). After blocking in 5% non-fat milk for 1 h at room temperature, the membranes were incubated overnight at 4 °C with primary antibodies against proliferating cell nuclear antigen (PCNA), ki-67, β-actin, Bax, Bcl-2, Wnt Family Member 1 (WNT1), β-catenin, C-myc, E-cadherin. Following this, the membranes were further probed for 1 h at room temperature with horseradish peroxidase (HRP)-conjugated secondary antibody. At last, specific protein signals were enhanced using Clarity Max™ Western ECL Substrate (Bio-Rad, Hercules, CA, USA). All antibodies were purchased from Abcam (Cambridge, UK).

### CCK-8 assay

HA-VSMCs (about 10,000 per well) were seeded into 96-well plates overnight. At 0, 24, 48, or 72 h post-transfection, 10 μl Cell Counting Kit-8 solution (MedChem Express, Monmouth Junction, NJ, USA) was added into each well of 96-well plates, followed by further incubation of 3 h at 37 °C. Then the absorbance was determined at the wavelength of 450 nm to assess cell proliferation.

### Colony formation assay

Transfected HA-VSMCs were seeded in a 12-well plate and cultured for 15 days in complete medium. Then cells were fixed with methanol and stained with 0.1% crystal violet solution (Sigma-Aldrich). At last, the total number of colonies with a minimum of 50 cells was detected using a microscope.

### Apoptotic cell percentage determination

Cell apoptotic percentage was tested using AnnexinV-FITC/PI Apoptosis Detection Kit (Vazyme Biotech Co., Ltd., Nanjing, China) following the protocols of manufacturer. Briefly, collected cells were re-suspended in 100 μl 1× Binding buffer, followed by the addition of 5 μl AnnexinV-FITC and 5 μl PI staining solution. After mixing, treated cells were incubated for 10 min at room temperature in dark. Then the apoptotic cell percentage were measured via a flow cytometry (BD Biosciences, San Jose, CA, USA) after adding 400 μl 1× Binding buffer.

### Caspase 3 activity detection

Caspase 3 activity was measured with Caspase 3 Assay Kit (Colorimetric, Abcam) according to the protocols of manufacturer. Briefly, treated cells (5 × 10^6^) were re-suspended with 50 μl of chilled Cell Lysis Buffer, followed by the isolation of cell supernatant and the measurement of protein concentration. Then 50 μl of 2× Reaction Buffer (containing 10 mM DTT) and 5 μl of 4 mM DEVD-p-NA substrate (200 μM final concentration) were added into 50 μl of each sample. After incubation for 2 h, the absorbance was detected at the wavelength of 405 nm. The absorbance was normalized by control group.

### Subcellular fraction

Nuclear or cytoplasmic RNA in HA-VSMCs was separated and purified using a Cytoplasmic and Nuclear RNA Purification Kit (Norgen Biotek, Thorold, Canada) following the instructions of manufacturer. Then the expression patterns of H19, GAPDH and U6 in nuclear and cytoplasm fractions were respectively determined by RT-qPCR assay.

### Luciferase activity assay

Partial fragments of H19 and WNT1 3’ UTR containing miR-148b binding sites were amplified by PCR and constructed into psiCHECK-2 vector (Promega, Madison, WI, USA) to produce H19-WT and WNT1-WT reporters. Also, Quickchage Multi Site-Directed Mutagenesis kit (Stratagene, Lajolla, CA, USA) was used to generate H19-MUT and WNT1-MUT reporters containing mutant miR-148b binding sites. Then constructed luciferase reporters were respectively transfected into HA-VSMCs together with miRNAs or along with plasmids. After 48 h, luciferase activities in cells were detected via a dual luciferase reporter assay kit (Promega) according to the protocols of manufacturer.

### RNA immunoprecipitation (RIP) assay

RIP assay were performed using EZ-Magna RIP kit (Millipore, Billerica, MA, USA) and Argonaute 2 (Ago2) antidody (Abcam) to explore whether H19 was existed in RNA induced silencing complex (RISC). Briefly, HA-VSMC cells were lysed in RIP lysis buffer, followed by the incubation of protein A/G magnetic beads and antibody against IgG (Millipore) or Ago2 (Abcam). Then RNAs in magnetic beads-binding complexes were purified. At last, RT-qPCR assay was employed to measure the enrichment patterns of H19 and miR-148b by IgG antibody or Ago2 antibody.

### Statistical analysis

All data were presented as mean ± SEM from at least three times independent assay. Student’s *t*-test or one-way variance analysis was used to investigate difference of data in different groups. *P*-values of less than 0.05 presented the difference was statistically significant.

## Results

### The level of H19 was increased and miR-148b was decreased in human AS patient serums and ox-LDL-stimulated HA-VSMCs

**Fig. 1:**
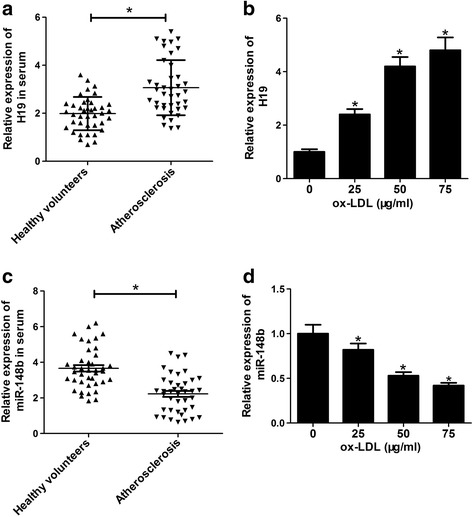
The level of H19 was increased and miR-148b was decreased in human AS patient serums and ox-LDL-stimulated HA-VSMCs. Expressions of H19 (**a**) and miR-148b (**c**) in the serums of human AS patients (*n* = 40) and healthy volunteers (n = 40). HA-VSMCs were treated with ox-LDL (0, 25, 50, 75 μg/ml) for 24 h, followed by the measurement of H19 (**b**) and miR-148b (**d**) expressions. Data are presented as mean ± SEM (*n* = 3) of at three independent experiments. *P < 0.05

Firstly, the expression patterns of H19 and miR-148b were investigated in human AS patient serums and ox-LDL-stimulated HA-VSMCs. RT-qPCR assay results revealed that the level of H19 was markedly increased (Fig. 1a) and miR-148b was strikingly decreased (Fig. 1c) in human AS patient serums (*n* = 40) than that in healthy volunteer serums (n = 40). Moreover, ox-LDL stimulation resulted in an increase of H19 expression (Fig. 1b) and a decrease of miR-148b expression (Fig. 1d) in HA-VSMCs in a dose-dependent manner. These results indicated H19 and miR-148b may be important mediators in AS progression.

### Knockdown of H19 suppressed proliferation and induced apoptosis in ox-LDL- stimulated HA-VSMCs

**Fig. 2:**
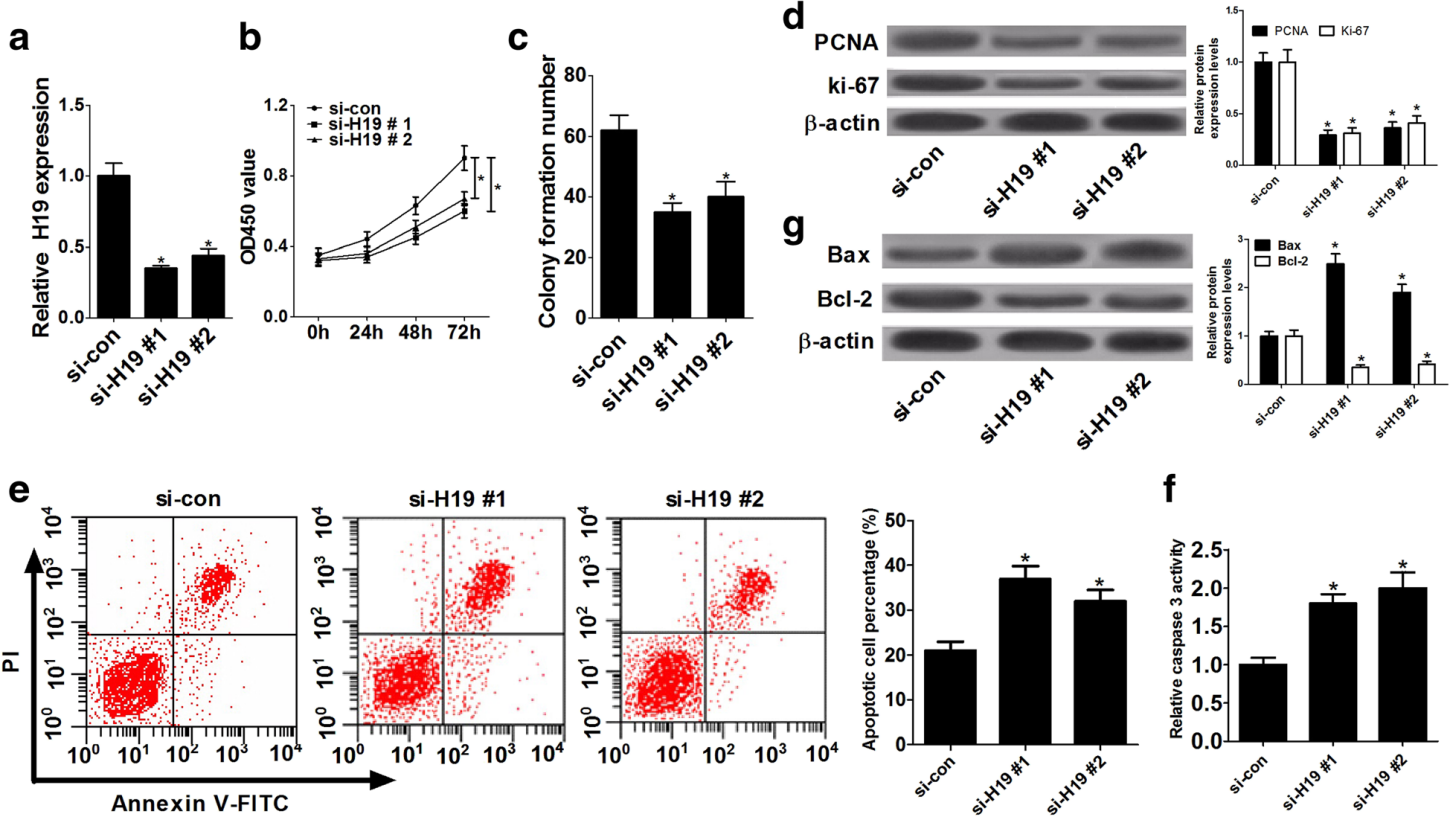
Knockdown of H19 suppressed proliferation and induced apoptosis in ox-LDL-stimulated HA-VSMCs. HA-VSMCs transfected with si-con, si-H19#1 or si-H19#2 were treated with 50 μg/ml ox-LDL for 24 h, followed by the detection of H19 expression (**a**), proliferative capacity (**b**), clone formation ability (**c**), proliferation marker (PCNA and ki-67) expressions (**d**), apoptotic cell percentage (**e**), caspase-3 activity (**f**), and apoptosis-related protein (Bcl-2 and Bax) expressions (**g**). Data are presented as mean ± SEM (*n* = 3) of at three independent experiments.*P < 0.05

Firstly, to explore the function of H19 in AS progression, we investigated the effects of H19 on proliferation and apoptosis in ox-LDL-stimulated HA-VSMCs by interfering H19 expression via siRNAs of H19 (si-H19#1 and si-H19#2). HA-VSMCs transfected with si-H19#1 or si-H19#2 were treated with 50 μg/ml ox-LDL for 24 h, followed by measurement of cell proliferative ability and apoptotic rate. RT-qPCR results showed that introduction of si-H19#1 and si-H19#2 markedly suppressed H19 expression compared with control siRNA (si-con) in ox-LDL-stimulated HA-VSMCs (Fig. 2a). CCK-8 assay revealed that depletion of H19 resulted in a notable inhibition of cell proliferation compared with scramble control in ox-LDL-stimulated HA-VSMCs (Fig. 2b). Moreover, colony formation assay also demonstrated that H19 knockdown significantly curbed colony formation potential of HA-VSMCs in comparison with negative control (Fig. 2c). Furthermore, the expressions of proliferative indicators (PCNA and ki-67) were detected in ox-LDL-stimulated HA-VSMCs. As expected, an apparent decrease of PCNA and ki-67 expression was observed in HA-VSMCs following H19 down-regulation (Fig. 2d). Additionally, flow cytometry assay showed that the apoptotic cell percentage was markedly increased in HA-VSMCs transfected with H19 siRNAs (si-H19#1, si-H19#2) than that in si-con-transfected HA-VSMCs (Fig. 2e). Consistently, as presented in Fig. 2f and g, loss of H19 resulted in a remarkable enhancement of caspase-3 activity, a marked increase of pro-apoptosis protein Bax expression, and a noteworthy reduction of anti-apoptosis protein Bcl-2 expression in HA-VSMCs when compared with negative control. In summary, these results suggested that H19 deficiency hampered proliferation and facilitated apoptosis in ox-LDL-stimulated HA-VSMCs.

### H19 suppressed miR-148b expression by direct interaction

**Fig. 3:**
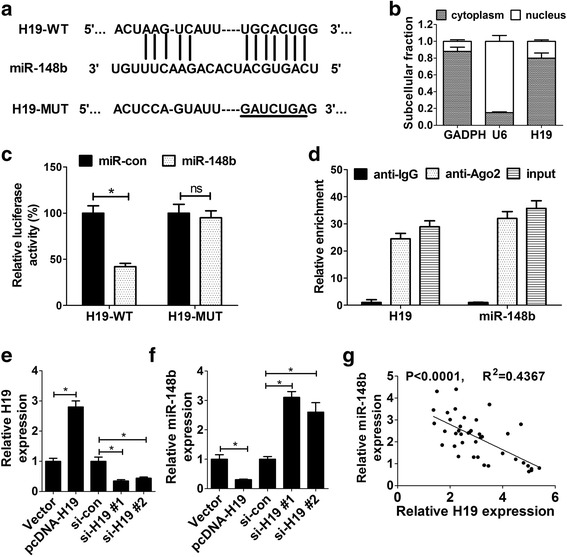
H19 suppressed miR-148b expression by direct interaction. **a** The putative binding sites between H19 and miR-148b and the mutant sites in H19-MUT reporter were displayed. **b** The expression patterns of GAPDH, U6 and H19 in cytoplasm and nucleus of HA-VSMCs were measured. GAPDH acted as a cytoplasm transcript control and U6 served as a nucleus transcript control. The data were displayed as a percentage of GAPDH, U6 and H19 in cytoplasm and nucleus. The total levels were standardized to be 1. **c** Luciferase activity was detected in HA-VSMCs co-transfected with H19-WT or H19-MUT reporter and miR-148b or its scramble control (miR-con). **d** RIP and RT-qPCR assays were performed to explore the binding efficiency of H19 and miR-148b to Ago2 protein in HA-VSMCs. **e** and **f** HA-VSMCs were transfected with pcDNA3.1 empty vetor, pcDNA-H19 overexpression plasmid, si-con, si-H19#1 or si-H19#2, followed by the detection of H19 **e** and miR-184 **f** levels at 48 h after transfection. **g** Correlation analysis of H19 and miR-148b expressions in 40 cases of AS patient serums. Data are presented as mean ± SEM (*n* = 3) of at three independent experiments.*P < 0.05

To further investigate molecular mechanisms of H19 in modulating progression of HA-VSMCs, miRcode online website was used to identify potential miRNA targets of H19. The results showed that there existed some complementary sites between H19 and miR-148b (Fig. 3a). A previous study revealed that miR-148b overexpression suppressed proliferation and migration of HA-VSMCs through directly targeting HSP90 in AS [22]. Hence, we intended to further explore whether miR-148b could directly interact with H19. Subcellular fraction assay revealed that H19 was mainly located in cytoplasm of HA-VSMCs, indicating a spatial possibility of interplay between H19 and miR-148b (Fig. 3b). To further demonstrate this conjecture, constructed H19-WT or H19-MUT luciferase reporter was transfected into HA-VSMC cells together with miR-148b or its scramble control (miR-con). Subsequent luciferase assay manifested that introduction of miR-148b caused a more than 50% loss of luciferase activity in H19-WT reporter compared with scramble control (Fig. 3c). However, miR-148b had no effect on luciferase activity of H19-MUT reporter in which the putative binding sites between H19 and miR-148b were mutant (Fig. 3c). To further demonstrate direct binding of H19 and miR-148b, RIP assay were performed using Ago2 antibody to explore whether H19 and miR-148b were involved in RNA-induced silencing complex (RISC). As shown in Fig. 3d, H19 and miR-148b were substantially enriched by Ago2 antibody compared with control IgG antibody, suggesting H19 and miR-148b were present in RISC. Taken together, these data manifested that H19 and miR-148b could combine with each other in HA-VSMCs. Next, we evaluate the regulatory effect of H19 on miR-148b expression by RT-qPCR assay. As displayed in Fig. 3e and f, transfection of pcDNA-H19 led to a striking increase of H19 expression and a significant decrease of miR-148b expression, while introduction of si-H19 led to a decline of H19 expression and an elevation of miR-148b expression. Furthermore, miR-148b expression was found to be inversely correlated with H19 level in 40 cases of AS patient serums (Fig. 3g). Based on these data, we drew a conclusion that H19 suppressed miR-148 expression by direct interaction.

### miR-148b inhibitor undermined the effects of H19 knockdown on proliferation and apoptosis of ox-LDL-stimulated HA-VSMCs

**Fig. 4:**
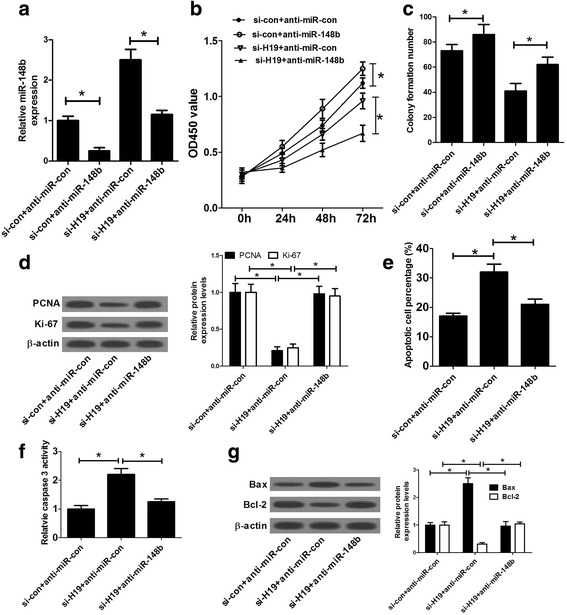
miR-148b inhibitor reversed the effects of H19 knockdown on proliferation and apoptosis in HA-VSMCs. (**a-c**) HA-VSMCs transfected with si-con+anti-miR-con, si-con+anti-miR-148b, si-H19 + anti-miR-con, or si-H19 and anti-miR-148b were stimulated with 50 μg/ml ox-LDL for 24 h, followed by the measurement of miR-148b level (**a**), cell proliferative capability (**b**), colony formation ability (**c**). **d-g** HA-VSMCs transfected with si-con+anti-miR-con, si-H19 + anti-miR-con, or si-H19 and anti-miR-148b were stimulated with 50 μg/ml ox-LDL for 24 h, followed by the determination of PCNA and Ki-67 expressions (**d**), apoptotic cell percentage (**e**), caspase-3 activity (**f**), Bax and Bcl-2 expressions (**g**). Data are presented as mean ± SEM (n = 3) of at three independent experiments. *P < 0.05

To further demonstrate whether H19-knockdown-triggered anti-proliferation and pro-apoptosis effects were mediated by miR-148b, we disturbed miR-148b expression in si-H19-transfected HA-VSMCs prior to ox-LDL stimulation. At first, RT-qPCR assay manifested that introduction of miR-148b inhibitor reduced miR-148b expression and weakened si-H19-mediated propulsive effect on miR-148b expression in ox-LDL-stimulated HA-VSMCs (Fig. 4a). Moreover, CCK-8 and colony formation assays revealed that the deficiency of miR-148b resulted in a marked enhancement of cell proliferative capacity and a notable increase of colony formation ability in ox-LDL-stimulated HA-VSMCs (Fig. 4b and c). In other words, miR-148b downregulation contributed to the proliferation of HA-VSMCs. Then CCK-8, colony formation and western blot assays further showed that the inhibitory effect of H19 deficiency on cell growth was greatly abrogated by miR-148b inhibitor, as presented by the enhancement of cell proliferative capacity (Fig. 4b), the increase of colony formation ability (Fig. 4c) and the upregulation of proliferative marker (PCNA and Ki-67) expressions (Fig. 4d) after down-regulating miR-148b in si-H19-transfected HA-VSMCs. As expect, repression of miR-148b expression markedly abated si-H19-induced apoptosis, revealed by the decrease of apoptotic cell percentage (Fig. 4e), the decline of caspase-3 activity and Bax expression (Fig. 4f and g), as well as the increase of Bcl-2 expression (Fig. 4g) in si-H19-transfected HA-VSMCs following miR-148b suppression. Taken together, these results indicated that H19-knockdown-triggered anti-proliferation and pro-apoptosis effect was mediated by miR-148b in ox-LDL-stimulated HA-VSMCs.

### WNT1 was a target of miR-148b

**Fig. 5:**
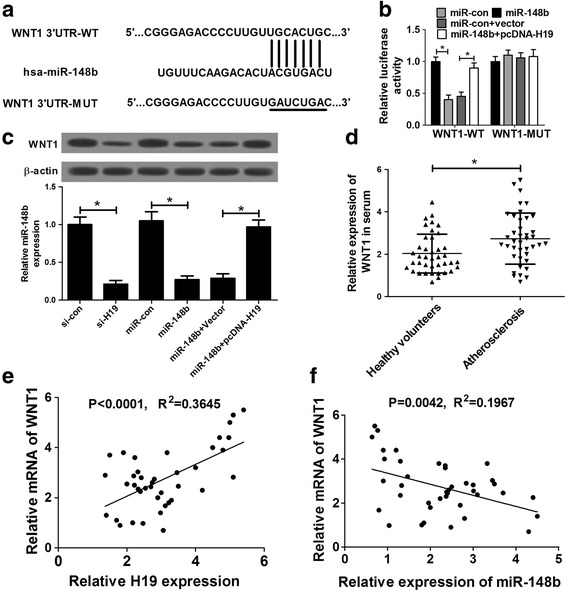
WNT1 was a target of miR-148b. **a** The predicted binding sequences between miR-148b and WNT1 3’UTR region, together with mutant sites in the WNT1-MUT reporter. **b** Luciferase activity was detected 48 h after transfection in HA-VSMCs co-transfected with WNT1-WT or WNT1-MUT reporter and miR-con, miR-148b, miR-con+pcDNA3.1 empty vector, or miR-148b + pcDNA-H19. **c** The effects of H19 knockdown (si-H19), miR-148b forced expression (miR-148b), and H19 overexpression (pcDNA-H19) on WNT1 protein expression were assessed using western blot assay at 48 h after transfection in HA-VSMCs. **d** WNT1 expression in serums of healthy volunteers (*n* = 40) and AS patients (n = 40). **e** and **f** The correlation analysis between WNT1 and H19 or miR-148b in 40 cases of AS patient serums. Data are presented as mean ± SEM (n = 3) of at least three independent experiments. *P < 0.05

A wealth of studies support that lncRNAs can act as competing endogenous RNAs (ceRNAs) of miRNAs to regulate expressions of target mRNAs [23, 24]. Hence, TargetScan software was used to decipher the potential targets of miR-148b. As displayed in Fig. 5a, WNT1 3’UTR region was found to contain some potential binding sequences of miR-148b. The following luciferase assay demonstrated that enforced expression of miR-148b markedly suppressed the luciferase activity of WNT1-WT reporter, while this effect of miR-148b was attenuated by overexpressing H19 in HA-VSMCs (Fig. 5b). However, no change was observed in the luciferase activity of WNT1-MUT reporter after miR-148b overexpression or H19 up-regulation (Fig. 5b). Western blots assay further showed that either H19 knockdown or miR-148b overexpression prominently inhibited WNT1 expression compared with their counterparts (Fig. 5c). Additionally, ectopic expression of H19 relieved miR-148b-mediated suppression on WNT1 expression in HA-VSMCs (Fig. 5c). Clinical data also revealed that WNT1 was highly expressed in serums (*n* = 40) of AS patients relative to that in healthy volunteers (Fig. 5d). Furthermore, RT-qPCR assay revealed that WNT1 expression was positively correlated with H19 expression (Fig. 5e), but was negatively correlated with miR-148b expression (Fig. 5f) in 40 cases of AS patient serums. Collectively, these data indicated that H19 acted as a ceRNA of miR-148 to enhance expression of target gene WNT1 in HA-VSMCs.

### miR-148b impeded proliferation and enhanced apoptosis by regulating WNT/β-catenin signaling in ox-LDL-stimulated HA-VSMCs

**Fig. 6:**
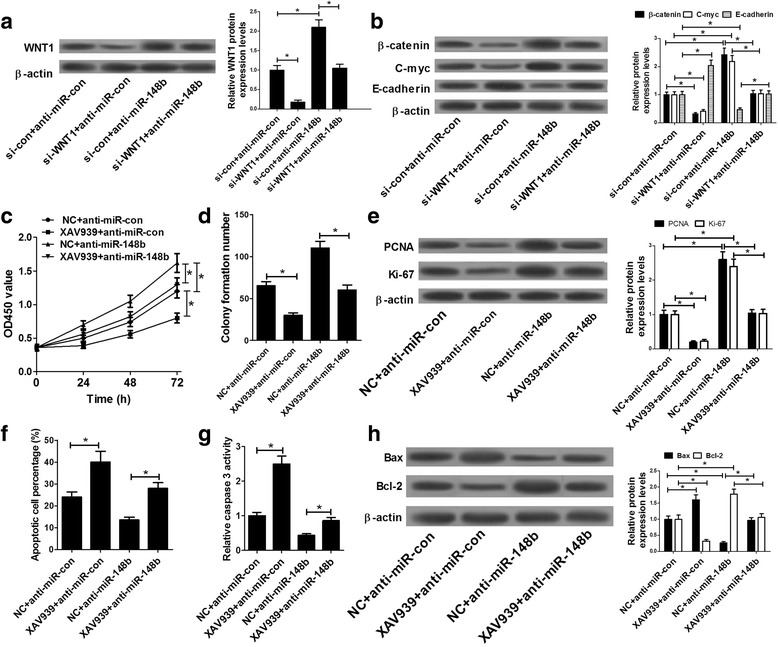
miR-148b modulated proliferation and apoptosis through WNT/β-catenin signaling in HA-VSMCs. (**a** and **b**) HA-VSMCs transfected with si-con+anti-miR-con, si-WNT1 + anti-miR-con, si-con+anti-miR-148b or si-WNT1 + anti-miR-148b were stimulated with 50 μg/ml ox-LDL for 24 h, followed by the evaluation of WNT1, β-catenin, C-myc and E-cadherin expressions. (C-H) HA-VSMCs transfected with anti-miR-con or anti-miR-148b were treated with or without XAV939 (10 μM) for 10 h and then stimulated with 50 μg/ml ox-LDL for 24 h, followed by the determination of cell proliferative ability (**c**), colony formation capability (**d**), PCNA and ki-67 expressions (**e**), apoptotic cell percentage (**f**), caspase-3 activity (**g**), as well as Bax and Bcl-2 expressions (**h**). Data are presented as mean ± SEM (n = 3) of at three independent experiments.*P < 0.05

WNT1 is a member of WNT family and WNT signaling has been identified as a vital regulating pathway in AS progression [25]. Thus, we aimed to further inspect the effects of WNT1 and miR-148b on WNT signaling pathway in ox-LDL-stimulated HA-VSMCs. Western bolt assay revealed that introduction of si-WNT1 generated an obvious decrease of WNT1 expression, and WNT1 knockdown markedly attenuated anti-miR-148b-mediated increase on WNT1 expression in HA-VSMCs (Fig. 6a). Next, we further explored the effect of si-WNT1 on the expressions of WNT signaling downstream genes (β-catenin, C-myc, E-cadherin) in HA-VSMCs. Results showed that WNT1 depletion markedly suppressed the expressions of β-catenin and C-myc as well as promoted E-cadherin expression in ox-LDL-stimulated HA-VSMCs, and anti-miR-148b-induced increase of β-catenin and C-myc expressions, and decrease of E-cadherin expression were substantially abated after downregulating WNT1 (Fig. 6b). These data indicated the regulatory effect of miR-148b on WNT/β-catenin signaling.

Next, we further discussed whether miR-148b exerted regulatory roles via WNT/β-catenin pathway in ox-LDL-stimulated HA-VSMCs. HA-VSMCs transfected with miR-con or miR-148b were treated with or without 10 μM XAV939 (a β-catenin inhibitor) for 10 h, followed by stimulated with 50 μg/ml ox-LDL for 24 h. The results showed that treatment with XAV939 remarkably suppressed cell proliferative ability (Fig. 6c), colony formation capacity (Fig. 6d) and proliferative marker (PCNA and ki-67) expressions (Fig. 6e). Conversely, downregulation of miR-148b facilitated cell growth, while the effect was markedly weakened following XAV939 treatment (Fig. 6c-e). Moreover, XAV939 strikingly enhanced apoptosis of HA-VSMCs, presented by the increase of apoptotic rate, caspase-3 activity and Bax expression, together with the decrease of Bcl-2 expression (Fig. 6f-h). Nevertheless, miR-148b inhibition lowered apoptosis of HA-VSMCs, whereas XAV939 treatment blocked anti-miR-148b-mediated anti-apoptosis effect (Fig. 6f-h). In a word, these results demonstrated that miR-148b exerted its anti-proliferation and pro-apoptosis effects by regulating WNT/β-catenin signaling in ox-LDL-stimulated HA-VSMCs.

## Discussion

Although much progress has been made in the pathogenesis and therapeutic options of AS, AS is still a major threaten of human health with a high morbidity and mortality worldwide [26]. Accumulating evidence shows that noncoding RNAs including lncRNAs and miRNAs play vital roles in the progression of AS [27].

Previous studies showed that H19 expression was elevated in human atherosclerotic lesions and rat VSMCs after injury [14, 15], indicating H19 was implicated in the development of AS. To further investigate the molecular mechanism of H19, miRcode online website was employed to figure out potential targets of H19. The results showed that miR-148b might interact with H19. miR-148b is a member of miR-148/miR-152 family, which also contains miR-148a and miR-152 [28]. Wu et al. previously confirmed that miR-152 suppressed proliferation and migration of human umbilical vein endothelial cells (HUVECs) by targeting ADAM17 [29]. Yang et al. demonstrated that miR-148a and miR-152 were associated with homocysteine-facilitated foam cell differentiation and atherosclerotic lesion [30]. Moreover, Zhang et al. verified that miR-148b overexpression blocked proliferation and migration of VSMCs through directly targeting HSP90 in AS [22].

Ox-LDL can induce AS by multiple mechanisms such as promoting ECs activation and dysfunction, VSMCs behavior, and foam cell formation [6, 31]. VSMC, a major cell type in blood vessels, participates in remodelling of arterial wall in order to maintain blood flow in affected vessels due to AS changes [32, 33]. Hence, in the present study, we aimed to investigate whether H19 was involved in the regulation of AS progression by modulating miR-148a in ox-LDL-stimulated HA-VSMCs.

We firstly demonstrated that the level of H19 was increased and miR-148b was decreased in AS patient serums. Moreover, we further validated the increase of H19 expression and decrease of miR-148b expression in HA-VSMCs treated with ox-LDL. Loss-of-function experiments showed that H19 knockdown resulted in a dramatic suppression of proliferation as well as a marked enhancement of apoptosis in ox-LDL-stimulated HA-VSMCs. Similarly, a previous report discovered that H19 overexpression facilitated proliferation and lowered apoptosis of VSMCs via regulation of MAPK and NF-kB signaling pathways [16]. H19 was also documented to stimulate VSMCs proliferation in a miR-675-dependent manner in the context of restenosis [34].

The following bioinformatics analysis, subcellular fraction assay, luciferase assay, RIP and RT-qPCR assays further demonstrated that H19 could bind to miR-148b to interfere its expression. Moreover, miR-148b expression was inversely correlated with H19 expression in AS patient serums. Hence, we proceeded to explore whether the effects of H19 on proliferation and apoptosis were mediated by miR-148b in ox-LDL-stimulated HA-VSMCs. Results indicated that H19 knockdown-mediated anti-proliferation and pro-apoptosis effects were greatly disrupted following miR-148b downregulation in ox-LDL-stimulated HA-VSMCs.

Emerging evidence shows that miRNAs exert functions by regulating translation or stability of target mRNAs. Hence, TargetScan software was used to search for potential target mRNAs of miR-148b. The results indicated that WNT1 was a potential target of miR-148b, which was validated by the subsequent luciferase assay. Moreover, WNT1 as a target of miR-148b has been demonstrated in hepatocellular cancer [21]. Our study further manifested that H19 acted as a ceRNA of miR-148b to enhance target gene WNT1 expression in HA-VSMCs. Additionally, WNT1 mRNA expression was positively correlated with H19 expression, but was negatively associated with miR-148b expression in AS patient serums.

WNT1 is a member of WNT family and WNT signaling has been shown as vital regulating pathways in the development of AS [25]. Studies also manifested that WNT1 induced the activation of β-catenin signaling and promoted cyclin D1 expression in VSMCs [35]. C-myc, a downstream target of Wnt signaling [36], promoted proliferation and suppressed apoptosis in rat VSMCs [37]. Also, activation of WNT signaling downregulated E-cadherin expression [38]. Moreover, a previous review has summarized the vital roles of cadherin-catenin complex and Wnt/β-catenin signaling pathway in VSMC progression [39]. Hence, the effects of WNT1 and miR-148b on the expressions of WNT signaling related genes (β-catenin, C-myc and E-cadherin) were measured in ox-LDL-stimulated HA-VSMCs. Results showed that WNT1 knockdown blocked WNT/β-catenin signaling, while miR-148b downregualtion activated WNT/β-catenin pathway in ox-LDL-stimulated HA-VSMCs. Moreover, the inductive role of anti-miR-148b on WNT/β-catenin signaling was abolished following WNT1 suppression. These results elucidated that inhibition of miR-148b stimulated WNT/β-catenin signaling via regulating WNT1. Next, we analyzed whether miR-148b affected proliferation and apoptosis through WNT/β-catenin pathway in ox-LDL-stimulated HA-VSMCs. The results indicated miR-148b deficiency caused a promotion of proliferative capacity and a repression of apoptosis in ox-LDL-stimulated HA-VSMCs, however, these effects were markedly reversed after treatment with XAV939. In a word, suppression of miR-148b contributed to growth and relieved apoptosis in ox-LDL-stimulated HA-VSMCs by modulating WNT/β-catenin signaling. Similarly, a previous document elaborated that miR-148b overexpression suppressed proliferation and migration of VSMCs through directly targeting HSP90 in AS [22].

## Conclusion

In summary, our study demonstrated that H19 depletion hampered proliferation and induced apoptosis in ox-LDL-stimulated HA-VSMCs by inactivating WNT/β-catenin signaling via miR-148b. This finding elucidated the involvement of H19/miR-148b/WNT/β-catenin regulatory axis in HA-VSMCs proliferation, hinting the potential application of H19 in preventing AS. However, the function and mechanisms of H19 in depth in the context of VSMCs and AS are still imperative to be discussed using animal models in the future.

## Abbreviations

AS: Atherosclerosis
ceRNA: Competing endogenous RNA
CVD: Cardiovascular diseases
Ecs: Endothelial cells
HA-VSMCs: Human aorta vascular smooth muscle cells
HUVECs: Human umbilical vein endothelial cells
lncRNAs: Long non-coding RNAs
ox-LDL: Oxidized low-density lipoprotein
PCNA: Proliferating cell nuclear antigen
RISC: RNA induced silencing complex
SMCs: Smooth muscle cells
VSMCs: Vascular smooth muscle cells
